# A Novel Nested Configuration Based on the Difference and Sum Co-Array Concept

**DOI:** 10.3390/s18092988

**Published:** 2018-09-07

**Authors:** Zhenhong Chen, Yingtao Ding, Shiwei Ren, Zhiming Chen

**Affiliations:** School of Information and Electronics, Beijing Institute of Technology, 5 South Zhongguancun Street, Haidian District, Beijing 100081, China; 3120140306@bit.edu.cn (Z.C.); ytd@bit.edu.cn (Y.D.); renshiwei@bit.edu.cn (S.R.)

**Keywords:** array signal processing, sparse array, degree of freedom, virtual array, DOA estimation

## Abstract

Recently, the concept of the difference and sum co-array (DSCa) has attracted much attention in array signal processing due to its high degree of freedom (DOF). In this paper, the DSCa of the nested array (NA) is analyzed and then an improved nested configuration known as the diff-sum nested array (DsNA) is proposed. We find and prove that the sum set for the NA contains all the elements in the difference set. Thus, there exists the dual characteristic between the two sets, i.e., for the difference result between any two sensor locations of the NA, one equivalent non-negative/non-positive sum result of two other sensor locations can always be found. In order to reduce the redundancy for further DOF enhancement, we develop a new DsNA configuration by moving nearly half the dense sensors of the NA to the right side of the sparse uniform linear array (ULA) part. These moved sensors together with the original sparse ULA form an extended sparse ULA. For analysis, we provide the closed form expressions of the DsNA locations as well as the DOF. Compared with some novel sparse arrays with large aperture such as the NA, coprime array and augmented nested array, the DsNA can achieve a higher number of DOF. The effectiveness of the proposed array is proved by the simulations.

## 1. Introduction

The direction-of-arrival (DOA) estimation is an important topic in many applications such as radar and sonar [1,2,3,4,5,6,7,8,9,10]. Many traditional high-resolution subspace-based estimators [11,12], which utilize the uniform linear array (ULA) as the array model, have been proposed for direction finding. As the degree of freedom (DOF) is limited by the array aperture, such estimators can detect no more than R−1 sources by using *R* physical sensors. In order to enhance the detection ability, many novel methods, such as the spatial smoothing based MUSIC (SS MUSIC) method [13], apply the concept of the Khatri–Rao (KR) product to sparse arrays for constructing the difference co-array (DCa) [13,14,15,16,17,18]. The combination of sparse arrays and the DCa concept can improve the DOF capacity and detect as many as $O(R^{2})$ sources.

The nested array (NA) [13] and coprime array (CA) [19], both of which consist of two uniform linear subarrays with different inter-element spacings, are two novel sparse arrays with high DOFs. In recent years, how to optimize these configurations to further increase the DOF has generated a new wave of interest. Since the CA has holes in its DCa, the corresponding improvements mainly focus on filling holes. An extended coprime array (ECA) developed by doubling the sensor number of one subarray was proposed in [20]. Compared with the CA, the ECA includes a larger consecutive range in its DCa since the increasement of the period of subarrays can help with filling holes. In [21,22], a coprime array with multi-period subarrays (CAMpS) was proposed by extending the two subarrays in the CA from one-period to multi-period. As the periodic extension version of the CA, the CAMpS can not only increase the DOF but also reduce the peak side-lobe. In [23], a generalized coprime array (GCA) comprised of two operations, which are the compression operation and displacement operation, was proposed. Since the two operations contribute to filling holes and enlarging the array aperture, the GCA can achieve a high number of DOF. Different from the CA, the NA has a hole-free DCa and requires less physical sensors to achieve the same DOF. However, since the NA contains a dense ULA, theoretically, its DOF can be further increased by redistributing sensors of its dense ULA part. In [24], an augmented nested array (ANA) concept was proposed. The ANA is constructed by splitting the dense ULA of the NA into several subarrays, which can be rearranged at the two sides of the sparse ULA of the NA. Thinning the dense ULA can reduce the redundancy of the DCa so that a higher number of DOF can be achieved.

**Figure 1:**
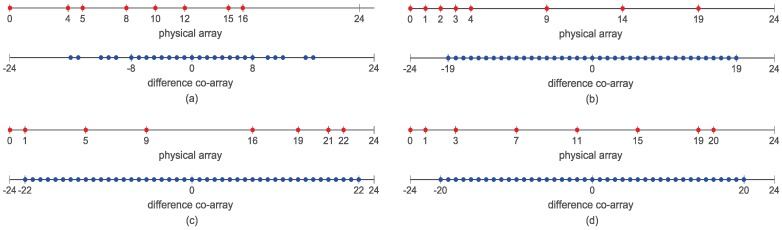
Examples of some DCa based sparse arrays with eight sensors and their corresponding difference co-arrays: (**a**) the coprime array; (**b**) the nested array; (**c**) the ANAII1; and (**d**) the ANAII2.

All the improved configurations mentioned above are developed based on the DCa concept. They can also be known as the DCa based sparse arrays. In order to make the relations of some traditional DCa based sparse arrays, such as the CA, NA and ANA, more clear, we give some examples of these arrays and their corresponding DCa in Figure 1. Note that ANAII1 and ANAII2 are two kinds of arrays with the highest DOF among the ANAs. It is obvious that combining sparse arrays and DCa concept can extend the aperture. Among all the arrays, the ANAs achieve the highest number of DOF. The NA has a larger aperture than the CA. All these arrays together with their improved configurations are pursuing a kind of sparser structure which can reduce the redundancy of the DCa. However, as shown in Figure 1, the DCa has a limitation, i.e., its DOF cannot be more than twice the physical aperture. Jointly utilizing the DCa and other co-arrays, such as the sum co-array [3,4,25,26,27], can break through the restriction and further extend the aperture.

In [3], we proposed a Vectorized Conjugate Augmented MUSIC (VCAM) estimator, which can construct a novel co-array known as the difference and sum co-array (DSCa). The DSCa consists of three parts, i.e., the difference, non-negative sum and non-positive sum co-arrays. Compared with the DCa, the DSCa has higher DOF and larger virtual aperture. Furthermore, the aperture of the DSCa can be more than twice the physical aperture, which could help to decrease the array size. However, Ref. [3] just summarizes some properties of the DSCa of the CA. The characteristics of the NA have not been studied.

In this paper, we first analyze the DSCa characteristics of the NA. From the properties, one can find that, for the NA, the redundancy of its DSCa is very high because the difference set is a subset of the sum set. That is to say, a difference virtual element generated by the difference result between any two physical sensor locations can always be replaced by the sum result of two other sensor locations. Such a dual characteristic provides a potential optimization strategy of the NA. As long as the original difference/sum result can be obtained, some physical sensors can be rearranged to reduce the redundancy of the virtual array and extend the array aperture. Based on this strategy, a diff-sum nested array (DsNA) is proposed by moving nearly half the dense sensors of the NA to the right side of the sparse uniform linear subarray. These moved sensors together with the original sparse ULA form an extended sparse ULA. The DsNA possesses the following advantages: (a) the DsNA has closed form expressions of the physical sensor locations and DOF; (b) the DSCa of the DsNA achieves reduced redundancy and larger aperture than that of the NA; (c) DsNA acquires a better DOA estimation performance and higher DOF than many other novel sparse arrays such as the CA and ANA. Extensive simulations verify the good performance of our proposed DsNA.

This paper is organized as follows. Section 2 reviews the VCAM algorithm. Section 3 derives the properties of the DSCa of the NA, introduces the DsNA and then analyzes its DSCa. Simulation results are provided in Section 4. Section 5 concludes this article.

Notations: In the paper, we utilize lowercase bold letters, such as a, to denote vectors. We utilize capital bold letters, such as A, to denote matrices. The sets are denoted by the capital outline letters, such as A. $\left\lceil a\right\rceil$ rounds a number to the nearest integer and $\left\lceil a\right\rceil \geq a$. ${\left(.\right)}^{*}$, ${\left(.\right)}^{T}$ and ${\left(.\right)}^{H}$ represent the conjugation, transpose and conjugate transpose of a matrix or vector, respectively. vec(.) represents the vectorization operation. ⊙ and ⊗ represent the Khatri–Rao product and the left Kronecker product, respectively.

## 2. Review of the VCAM Algorithm

In this paper, we exploit the VCAM estimator to construct the DSCa. It is noted that the VCAM method is originally developed for detecting pulsed radars whose waveforms are simple pulse [28,29,30]. This kind of radar is also known as simple pulse radar. The simple pulse radar signal is a sinusoidal waveform with deterministic amplitude, frequency and phase. Since the baseband signal frequency is a characteristic parameter of the pulsed radar, the baseband signal frequencies of different pulsed radars are different. Simple pulse radars are of good application value in many engineering projects. As a kind of ground-based surveillance radar, simple pulse radars are often used for remote search which would not demand high resolution. The sinusoidal waveforms of simple pulse radars are much easier to handle compared with some other kinds of pulsed radars, such as chirp radars. Simple pulse radars are also often used to assist chirp radars for velocity measurement. Due to the range doppler coupling effect, chirp radars could fail to measure velocity of target. In contrast, simple pulse radars would not be affected by the effect. Therefore, simple pulse and chirp pulse are often jointly utilized to estimate range and velocity in some engineering projects. The VCAM method can be applied to non-cooperative radar arrays to estimate directions of multiple simple pulse radars located at different positions. The VCAM algorithm and its data model are summarized below.

According to [3,25], we assume *Q* deterministic far-field plane wave sources impinging on the sensor array from directions $\left\{\theta_{1},\ldots ,\theta_{Q}\right\}$. The multipath effect is not considered in our model. The sensor location set is denoted as $D=\{d_{1},\ldots ,d_{R}\}$ where $d_{1}=0$ due to us selecting the first sensor as the reference. The *q*th $\left(1\leq q\leq Q\right)$ signal can be represented as $s_{q}\left(t\right)=A_{q}e^{j\omega_{q}t}$, where $A_{q}$ is the deterministic complex amplitude, $\omega_{q}$ is the baseband signal frequency and $\omega_{q}\neq \omega_{p}$ (1≤p≤Q,p≠q). Due to baseband signal frequencies of different radars being different, the signals are mutually orthogonal to each other. Then, the received signal can be represented as

(1) $$\begin{array}{c} x\left(t\right)=\sum_{q=1}^{Q}a(\theta_{q})s_{q}\left(t\right)+n\left(t\right)=\mathrm{As}\left(t\right)+n\left(t\right), \end{array}$$

where $s\left(t\right)={\left[s_{1}\left(t\right),s_{2}\left(t\right),\ldots ,s_{Q}\left(t\right)\right]}^{T}$ is the source signal vector, $n\left(t\right)={\left[n_{1}\left(t\right),n_{2}\left(t\right),\ldots ,n_{R}\left(t\right)\right]}^{T}$ is the zero-mean uncorrelated white complex Gaussian noise vector with variance $\sigma_{n}^{2}$, $A=[a\left(\theta_{1}\right),a\left(\theta_{2}\right),\ldots ,a\left(\theta_{Q}\right)]$ denotes the manifold matrix and $a\left(\theta_{q}\right)={\left[1,e^{j2\pi d_{2}\sin\left(\theta_{q}\right)/\lambda },\ldots ,e^{j2\pi d_{R}\sin\left(\theta_{q}\right)/\lambda }\right]}^{T}$ is the steering vector corresponding to the direction $\theta_{q}$. λ represents the signal wavelength. By collecting $N_{x}$ samples from the first sensor output $x_{1}\left(t\right)$ and the *r*th (1≤r≤R) output $x_{r}\left(t\right)$, we can obtain two vectors: $\left[x_{1}\left(1\right),x_{1}\left(2\right),\ldots ,x_{1}\left(N_{x}\right)\right]$ and $\left[x_{r}\left(1+\tau \right),x_{r}\left(2+\tau \right),\ldots ,x_{r}\left(N_{x}+\tau \right)\right]$, where τ≠0. Assuming $N_{x}$ is sufficiently large, one can obtain the following time average function

(2) $$\begin{array}{cc} R_{x_{1}^{*}x_{r}}\left(\tau \right) & =\frac{1}{N_{x}}\sum_{t=1}^{N_{x}}x_{1}^{*}\left(t\right)x_{r}\left(t+\tau \right)\\ & =\sum_{q_{1}=1}^{Q}\sum_{q_{2}=1}^{Q}\left\{a_{1}^{*}\left(\theta_{q_{1}}\right)a_{r}\left(\theta_{q_{2}}\right)R_{s_{q_{1}}^{*}s_{q_{2}}}\left(\tau \right)\right\}+R_{n_{1}^{*}n_{r}}\left(\tau \right), \end{array}$$

where $a_{1}^{*}\left(\theta_{q_{1}}\right)=1$, $a_{r}\left(\theta_{q_{2}}\right)=e^{j2\pi d_{r}\sin\left(\theta_{q_{2}}\right)/\lambda }$, Rsq1*sq2(τ)=∑t=1Nxsq1*(t)sq2(t+τ)sq2*(t)sq(t+τ)NxNx=Aq1*Aq2ejωq2τ∑t=1Nxej(ωq2−ωq1)t/Nx and $R_{n_{1}^{*}n_{r}}\left(\tau \right)=\sum_{t=1}^{N_{x}}n_{1}^{*}\left(t\right)n_{r}\left(t+\tau \right)/N_{x}\approx \sigma_{n}^{2}\delta \left(\tau \right)\delta \left(r-1\right)=0$. Due to ∑t=1Nxej(ωq2−ωq1)tej(ωq2−ωq1)tNxNx≈0 for $\omega_{q_{1}}\neq \omega_{q_{2}}$ ($q_{1}\neq q_{2}$), Equation (2) can be simplified as

(3) $$\begin{array}{c} R_{x_{1}^{*}x_{r}}\left(\tau \right)=\sum_{q=1}^{Q}e^{j2\pi d_{r}\sin\left(\theta_{q}\right)/\lambda }R_{s_{q}^{*}s_{q}}\left(\tau \right), \end{array}$$

where Rsq*sq(τ)=∑t=1Nxsq*(t)sq(t+τ)sq*(t)sq(t+τ)NxNx=|Aq|2ejωqτ has the same form as the source signal $s_{q}\left(t\right)=A_{q}e^{j\omega_{q}t}$. Thus, $R_{s_{q}^{*}s_{q}}\left(\tau \right)$ amounts to a signal source whose DOA is $\theta_{q},q=1,2,\ldots ,Q$ and power is $|A_{q}{|}^{4}$. Stacking all the vectors $R_{x_{1}^{*}x_{r}}\left(\tau \right),r=1,\ldots ,R$, we can obtain

(4) $$\begin{array}{c} v_{x}\left(\tau \right)={\left[R_{x_{1}^{*}x_{1}},R_{x_{1}^{*}x_{2}},\ldots ,R_{x_{1}^{*}x_{R}}\right]}^{T}=Av_{s}\left(\tau \right), \end{array}$$

where $v_{s}\left(\tau \right)={\left[R_{s_{1}^{*}s_{1}}\left(\tau \right),\ldots ,R_{s_{Q}^{*}s_{Q}}\left(\tau \right)\right]}^{T}=[{\left|A_{1}\right|}^{2}e^{j\omega_{1}\tau },\ldots ,{{\left|A_{Q}\right|}^{2}e^{j\omega_{Q}\tau }]}^{T}$. Then, we can further have ${\left[v_{x}\left(-\tau \right)\right]}^{*}=A^{*}v_{s}\left(\tau \right)$. It is obvious that $v_{x}\left(\tau \right)$ and ${\left[v_{x}\left(-\tau \right)\right]}^{*}$ share the same first row. Thus, for reducing the computation complexity, we eliminate the first row from ${\left[v_{x}\left(-\tau \right)\right]}^{*}$ to obtain ${\left[v_{x}^{\prime }\left(-\tau \right)\right]}^{*}={\left(A^{\prime }\right)}^{*}v_{s}\left(\tau \right)$, where $A^{\prime }=\left[a^{\prime }\left(\theta_{1}\right),\ldots ,a^{\prime }\left(\theta_{Q}\right)\right]$ with $a^{\prime }\left(\theta \right)={\left[e^{j2\pi d_{2}\sin\left(\theta \right)/\lambda },\ldots ,e^{j2\pi d_{R}\sin\left(\theta \right)/\lambda }\right]}^{T}$. Concatenating $v_{x}\left(\tau \right)$ and ${\left[v_{x}^{\prime }\left(-\tau \right)\right]}^{*}$ together yields the following conjugate augmented correlation vector

(5) $$\begin{array}{c} v\left(\tau \right)=\left[\begin{array}{c} v_{x}\left(\tau \right)\\ {\left[{v^{\prime }}_{x}\left(-\tau \right)\right]}^{*} \end{array}\right]=\tilde{A}v_{s}\left(\tau \right), \end{array}$$

where $\tilde{A}={\left[A^{T},{\left(A^{\prime }\right)}^{H}\right]}^{T}=\left[\tilde{a}\left(\theta_{1}\right),\ldots ,\tilde{a}\left(\theta_{Q}\right)\right]$ with $\tilde{a}\left(\theta_{q}\right)={\left[a{\left(\theta_{q}\right)}^{T},{\left(a^{\prime }\left(\theta_{q}\right)\right)}^{H}\right]}^{T}$. By choosing a set of different time lags, i.e., $\tau =\tau_{s},2\tau_{s},\ldots ,N_{\tau }\tau_{s}$, we can obtain the following pseudo-data matrix

(6) $$\begin{array}{c} V=\left[v\left(\tau_{s}\right),v\left(2\tau_{s}\right),\ldots ,v\left(N_{\tau }\tau_{s}\right)\right]=\tilde{A}\mathrm{BW}, \end{array}$$

where $N_{\tau }$ is the number of pseudo snapshots, $\tau_{s}$ is the pseudo sampling period and is set to satisfy the sampling theorem, $B=diag([|A_{1}{|}^{2},\cdots ,|A_{Q}{|}^{2}])$ and $W={\left[w^{T}\left(1\right),w^{T}\left(2\right),\ldots ,w^{T}\left(Q\right)\right]}^{T}$ with the *q*th row vector being $w\left(q\right)=\left[e^{j\omega_{q}\tau_{s}},e^{j\omega_{q}2\tau_{s}},\ldots ,e^{j\omega_{q}N_{\tau }\tau_{s}}\right]$. When $N_{\tau }$ is sufficiently large, $w(q_{1})$ could be considered to be orthogonal to $w(q_{2})$$\left(q_{2}\neq q_{1}\right)$, i.e., $w\left(q_{1}\right)w^{T}\left(q_{2}\right)/N_{\tau }\approx 0$. Therefore, by choosing a sufficiently large value for $N_{\tau }$, the covariance matrix of v(τ) can be estimated by

(7) $$R_{\mathrm{vv}}=\frac{1}{N_{\tau }}VV^{H}=\tilde{A}R_{v_{s}v_{s}}{\tilde{A}}^{H},$$

where $R_{v_{s}v_{s}}=B\left(WW^{H}/N_{\tau }\right)B^{H}=diag\left({\left|A_{1}\right|}^{4},\ldots ,{\left|A_{Q}\right|}^{4}\right)$. Vectorizing $R_{\mathrm{vv}}$ results in

(8) $$z=vec\left(R_{\mathrm{vv}}\right)=\left({\tilde{A}}^{*}⊙\tilde{A}\right)p,$$

where $p=\left[\right|A_{1}{|}^{4},\cdots ,{\left|A_{Q}{|}^{4}\right]}^{T}$ and the *q*th column vector of ${\tilde{A}}^{*}⊙\tilde{A}$ is

(9) $$\begin{array}{cc} {\tilde{a}}^{*}\left(\theta_{q}\right)⊗\tilde{a}\left(\theta_{q}\right) & ={\left[\begin{array}{c} a(\theta_{q})\\ {\left(a^{\prime }\left(\theta_{q}\right)\right)}^{*} \end{array}\right]}^{*}⊗\left[\begin{array}{c} a(\theta_{q})\\ {\left(a^{\prime }\left(\theta_{q}\right)\right)}^{*} \end{array}\right]\\ & =\left[\begin{array}{c} a^{*}\left(\theta_{q}\right)⊗a\left(\theta_{q}\right)\\ a^{*}\left(\theta_{q}\right)⊗{\left(a^{\prime }\left(\theta_{q}\right)\right)}^{*}\\ a^{\prime }\left(\theta_{q}\right)⊗a\left(\theta_{q}\right)\\ a^{\prime }\left(\theta_{q}\right)⊗{\left(a^{\prime }\left(\theta_{q}\right)\right)}^{*} \end{array}\right]. \end{array}$$

Comparing Equations (8) and (1), we can find that z behaves like the equivalent received signal at a virtual array whose manifold matrix is ${\tilde{A}}^{*}⊙\tilde{A}$. From Equation (9), it is obvious that any virtual sensor location can be represented as one of the following forms: $d_{r_{1}}-d_{r_{2}}$, $d_{r_{1}}+d_{r_{2}}$ and $-(d_{r_{1}}+d_{r_{2}})$ ($1\leq r_{1},r_{2}\leq R$). Thus, the obtained virtual array is a DSCa, which consists of the difference co-array, the non-negative sum co-array and the non-positive sum co-array. In order to solve the coherent issue of z, we apply the spatial smoothing algorithm, which requires that the virtual array be a ULA, to deal with z. Assume that the consecutive range of the DSCa is $\left[-L_{c}d,L_{c}d\right]$, where *d* is the unit inter-element spacing. Then, removing the repeated and discrete location lags in Equation (8), we obtain

(10) $$\begin{array}{c} \hat{z}=\hat{A}p, \end{array}$$

where $\hat{A}$ is a $(2L_{c}+1)\times Q$ manifold matrix corresponding to the virtual sensors located from $-L_{c}d$ to $L_{c}d$. Now, we divide the virtual ULA into $L_{c}+1$ subarrays, each of which contains $L_{c}+1$ elements. The elements of the *l*th $\left(l=1,\ldots ,L_{c}+1\right)$ subarray are located from (−l+1)d to $(-l+1+L_{c})d$. Then, we extract the rows, which correspond to the *l*th subarray, from $\hat{z}$ in Equation (10) to obtain the vector ${\hat{z}}_{l},l=1,\ldots ,L_{c}+1$. By applying MUSIC to the following full-rank covariance matrix

(11) $$\begin{array}{c} R_{\hat{z}\hat{z}}=\frac{1}{L_{c}+1}\sum_{l=1}^{L_{c}+1}{\hat{z}}_{l}{\hat{z}}_{l}^{H}, \end{array}$$

we can estimate the DOA of the signals.

**Remark** **1.** *Compared with the methods (e.g., SS MUSIC) which utilize the spatial information of the received signals to construct the DCa, the VCAM method has larger DOF capacity and improved DOA performance. The multi-frequency form of the signal model is a key for the better performance. It allows one to utilize not only the spatial information of received deterministic signals but also the temporal information. Thus, more additional information can be used for DOA estimation. Based on this advantage brought by the multi-frequency model, the VCAM method jointly utilizes both the temporal information and the spatial information to obtain the conjugate augmented correlation vector v(τ). This operation extends the array manifold matrix from A to $\tilde{A}$. Then, the covariance matrix of the conjugate augmented correlation vector has a larger dimension and contains more information, which allows the VCAM method to construct the DSCa consisting of the DCa and sum co-array. Therefore, compared with the DCa obtained by using spatial information, the DSCa obtained by jointly using the spatial and temporal information has higher DOF and larger virtual aperture. It should also be noted that the size of the temporal window used for observing the orthogonal deterministic signals depends on the frequency spacings of signals. Small frequency spacings result in a large observation window. For instance, if one want to let ∑t=1Nxej(ωq2−ωq1)tej(ωq2−ωq1)tNxNx≈0$\left(\omega_{q_{1}}\neq \omega_{q_{2}}\right)$, the value of $N_{x}$ will depend on $\left|\omega_{q_{2}}-\omega_{q_{1}}\right|$. They are inversely related. The effect of frequency spacings on observation window size is very noticeable when the frequency spacing is small, e.g., less than $n\times 10^{-2}$ Hz (0<n<10). However, when the frequency spacing is no less than $n\times 10^{-1}$ Hz, this kind of effect is no longer so obvious. No matter if the frequency interval is a few kilohertz or only a few hertz, the minimum observation temporal window size that makes the two signals approximately orthogonal is almost the same. In real systems, the baseband signal frequency spacing of different simple pulse radars has been actually large enough to ensure that the observation temporal window for the VCAM method does not need to be so large. Thus, when the temporal window size takes a relatively large value, the orthogonal characteristic can be guaranteed.*

*The VCAM method is originally applied to non-cooperative radar arrays to estimate directions of multiple simple pulse radars located at different positions. However, based on the discussion of the method, it is obvious that a cooperative setting is also one kind of suitable scenario for the VCAM method. In the cooperative setting, we have Q tags that can be instructed by a central control node to actively transmit mutually orthogonal sinusoidal waveforms with constant amplitude, frequency and phase. The goal is localizing the tags. One can choose a set of favorable frequencies for these Q sinusoidal signals to help improve the performance of our algorithm. Therefore, a cooperative setting can make full use of the superior performance and high DOF capacity of the VCAM algorithm.*

## 3. The Diff-Sum Nested Array Based on the Concept of the Difference and Sum Co-Array

In this section, we will first make the NA as the array model and analyze the properties of its DSCa, as well as the relationship between the consisting difference and sum co-arrays. Then, based on the summarized characteristic, we propose an improved nested configuration, i.e., the DsNA. Compared with the NA, the proposed configuration acquires larger aperture and has less redundancy in its DSCa so that it can achieve a higher number of DOF. For convenience, we define the following two operations: $\pm \left(A\pm B\right)=\left\{\pm \left(a\pm b\right)\left|a\in A,b\in B\right.\right\}$ and $\pm \left(A\pm c\right)=\left\{\pm \left(a\pm c\right)\left|a\in A\right.\right\}$, where A and B are two given sets, and *c* is a scalar.

### 3.1. The Properties of the DSCa of the NA

**Figure 2:**
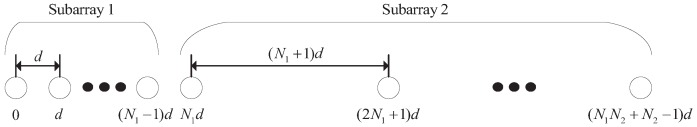
The nested array configuration.

As shown in Figure 2, the NA consists of two ULAs. Subarray 1 is a dense ULA including $N_{1}$ sensors with the inter-sensor spacing of one unit. Subarray 2 is a sparse ULA containing $N_{2}$ sensors with the inter-sensor spacing of $N_{1}+1$ units. The total sensor number of the NA is $N_{1}+N_{2}$. For convenience, we normalize all the locations by the unit inter-element spacing *d*. Then, the location set of the NA can be represented as $D_{NA}=D_{1}\cup D_{2}$, where $D_{1}=\left\{n_{1}\left|0\leq n_{1}\leq N_{1}-1\right.\right\}$ and $D_{2}=\left\{N_{1}+n_{2}\left(N_{1}+1\right)\left|0\leq n_{2}\leq N_{2}-1\right.\right\}$.

According to Equation (9), the DSCa can be represented as

(12) $$\begin{array}{c} \begin{array}{c} L_{ds}=L_{diff}\cup \underset{L_{sum}}{\underset{︸}{L_{sum}^{+}\cup L_{sum}^{-}}}, \end{array} \end{array}$$

where $L_{diff}=D-D$ is the difference set, $L_{sum}^{+}=D+D$ is the non-negative sum set, $L_{sum}^{-}=-\left(D+D\right)$ is the non-positive sum set, and $L_{sum}=L_{sum}^{+}\cup L_{sum}^{-}$ is the sum set. For the NA, Ref. [13] has concluded that its difference set $L_{diff}$ possesses all of the consecutive elements in the range $\left[-\left(N_{1}N_{2}+N_{2}-1\right),N_{1}N_{2}+N_{2}-1\right]$.

When using the NA in Figure 2 as the array model (i.e., $D=D_{NA},R=N_{1}+N_{2}$), $L_{sum}^{+}$ can be expressed as

(13) $$\begin{array}{c} \begin{array}{c} L_{sum}^{+}=\left\{D_{1}+D_{1}\right\}\cup \left\{D_{1}+D_{2}\right\}\cup \left\{D_{2}+D_{2}\right\}, \end{array} \end{array}$$

where $D_{1}+D_{1}=\left\{0,1,\ldots ,2\left(N_{1}-1\right)\right\}$, $D_{2}+D_{2}=\left\{2N_{1}+x\left(N_{1}+1\right)\left|0\leq x\leq 2(N_{2}-1)\right.\right\}$, and $D_{1}+D_{2}=\left\{N_{1},N_{1}+1,\ldots ,2N_{1}-1\right\}\cup \left\{2N_{1}+1,2N_{1}+2,\ldots ,3N_{1}\right\}\cup \cdots \cup \left\{N_{1}N_{2}+N_{2}-1,N_{1}N_{2}+N_{2},\ldots ,N_{1}N_{2}+N_{1}+N_{2}-2\right\}$. The holes in $D_{1}+D_{2}$ form the set $H_{1}=\left\{2N_{1}+x\left(N_{1}+1\right)|0\leq \right.\left.x\leq N_{2}-2\right\}$. It is obvious that $H_{1}\cup \left\{N_{1}N_{2}+N_{1}+N_{2}-1\right\}\subseteq \left\{D_{2}+D_{2}\right\}$. Thus, we can conclude that the consecutive range of $L_{sum}^{+}$ is $\left[0,N_{1}N_{2}+N_{1}+N_{2}-1\right]$. Since $L_{sum}^{-}$ is the flipped version of $L_{sum}^{+}$, we summarize the property of $L_{sum}$ as the following proposition.

**Proposition** **1.** *The consecutive range of the sum set $L_{sum}$ of the NA is $\left[-\left(N_{1}N_{2}+N_{1}+N_{2}-1\right),N_{1}N_{2}+N_{1}+N_{2}-1\right]$.*

Comparing the properties of $L_{diff}$ and $L_{sum}$, it is clear that $L_{diff}$ is a subset of $L_{sum}$, which means that, for any difference virtual element $d_{r_{1}}-d_{r_{2}}\left(d_{r_{1}}.d_{r_{2}}\in D,1\leq r_{1},r_{2}\leq R\right)$ in $L_{diff}$, there always exists one equivalent sum virtual element $d_{r_{3}}+d_{r_{4}}$ or $-\left(d_{r_{3}}+d_{r_{4}}\right)\left(d_{r_{3}}.d_{r_{4}}\in D,1\leq r_{3},r_{4}\leq R\right)$ in $L_{sum}$, i.e., $d_{r_{1}}-d_{r_{2}}=d_{r_{3}}+d_{r_{4}}$ or $-(d_{r_{3}}+d_{r_{4}})$. Such a dual characteristic reveals the high redundancy of the DSCa of the NA, but simultaneously can be used to rearrange the physical sensors for extending the array aperture and DOF. Thus, in order to derive an optimization strategy of the NA, we need to make a concrete analysis of the dual characteristic. First, we introduce the concept of the dual pair.

**Definition** **1.** *(Dual pair). For an arbitrary hole $d_{h}$ in the NA and two elements $d_{1D_{1}},d_{2D_{1}}\in D_{1}\cup \left\{N_{1}\right\}$, if there exist $d_{1D_{2}},d_{2D_{2}}\in D_{2}$ to make $d_{h}=d_{1D_{2}}-d_{1D_{1}}=d_{2D_{2}}+d_{2D_{1}}$, the pair $\left\{d_{1D_{1}},d_{2D_{1}}\right\}$ is called the dual pair.*

**Figure 3:**
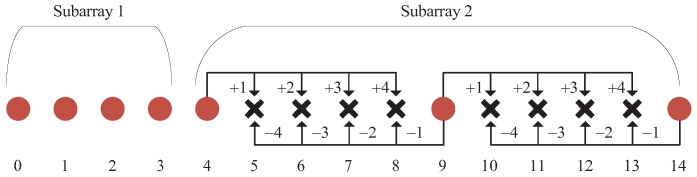
An example of filling the holes in the NA with $\left(N_{1},N_{2}\right)=\left(4,3\right)$.

From Definition 1, one can know that, when retaining all the elements (e.g., $d_{1D_{2}}$ and $d_{2D_{2}}$) in $D_{2}$, a specific hole $d_{h}$ in the NA can still be filled even if one element in the corresponding dual pair $\left\{d_{1D_{1}},d_{2D_{1}}\right\}$ is eliminated. To illustrate this characteristic clearly, we depict in Figure 3 how the holes in the NA with $\left(N_{1},N_{2}\right)=\left(4,3\right)$ can be filled by the difference or sum results of $D_{1}\cup \left\{N_{1}\right\}$ and $D_{2}$. Table 1 summarizes all the dual pairs in Figure 3. Take the hole location 6 for example. In the premise that $9,4\in D_{2}$ are retained in the sparse ULA, if 3 is removed, one can still use 4 + 2 to form 6. Similarly, if 2 is moved away, 9 − 3 can be used. Therefore, either element in the dual pair $\left\{3,2\right\}$ can be used to fill the hole location 6. It is noted that $\left\{3,2\right\}$ and $\left\{2,3\right\}$ are the same dual pair—so are $\left\{4,1\right\}$ and $\left\{1,4\right\}$. Figure 3 and Table 1 also show that the dual pairs used to fill holes between any two adjacent sensors of Subarray 2 are exactly the same. This property always holds whatever the sensor number $N_{2}$ is. Denote the number of dual pairs in NA as $N_{dual}$. In the following proposition, we provide the closed form expression of the dual pairs.

**Proposition** **2.** *For the NA with $\left(N_{1},N_{2}\right)$, the dual pairs are characterized by the set $P_{NA}=\left\{\left\{d_{1D_{1}},d_{2D_{1}}\right\}\left|1\leq d_{1D_{1}},d_{2D_{1}}\leq N_{1},d_{1D_{1}}+d_{2D_{1}}=N_{1}+1\right.\right\}$. When $N_{1}$ is odd, $P_{NA}=\left\{\left\{1,N_{1}\right\},\left\{2,N_{1}-1\right\},\ldots ,\right.\left.\left\{\left(N_{1}+1\right)/2,\left(N_{1}+1\right)/2\right\}\right\}$. The dual pair number is $N_{dual}=\left(N_{1}+1\right)/2$. When $N_{1}$ is even, the dual pair set becomes $P_{NA}=\left\{\left\{1,N_{1}\right\},\left\{2,N_{1}-1\right\},\ldots ,\left\{N_{1}/2,\left(N_{1}+2\right)/2\right\}\right\}$. In this case, $N_{dual}=N_{1}/2$.*

**Proof.**  See Appendix A. ☐

According to Proposition 2, one can find that the dual pair number is N1N122, which is approximately half the number of dense sensors in the NA. This proposition together with the previous analysis of dual pairs provide a potential strategy to improve the NA. Under the premise that all the elements in $D_{2}$ are retained, we can choose any $N_{c}$ ($N_{c}$$\leq N_{dual}$) dual pairs, take one element from each of these chosen pairs and move them to the positions $N_{1}+x\left(N_{1}+1\right)$, $x=N_{2},N_{2}+1,\ldots ,N_{2}+N_{c}-1$. Then, the remaining sensors in $D_{1}$ form a sparse array. The moved sensors together with Subarray 2 form an extended sparse ULA with sensors located at $Z=\left\{N_{1}+n_{2}\left(N_{1}+1\right)\left|0\leq n_{2}\leq N_{2}+N_{c}-1\right.\right\}$. As this extended sparse ULA is a periodic extension version of Subarray 2, the holes in Z can be filled by the difference or sum results of Z and the retained elements in $D_{1}\cup \left\{N_{1}\right\}$. It is noted that, in $P_{NA}$, $\left\{1,N_{1}\right\}$ is the only sensor pair that contains the element $N_{1}$ in $D_{2}$. We will always move 1 and retain $N_{1}$ when considering moving one element in $\left\{1,N_{1}\right\}$.

**Figure 4:**
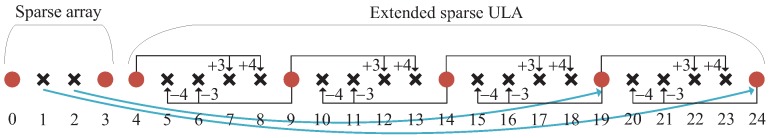
An example of rearranging dual pair elements of the NA with $\left(N_{1},N_{2}\right)=\left(4,3\right)$.

Figure 4 illustrates one moving scheme of the NA with $\left(N_{1},N_{2}\right)=\left(4,3\right)$. 2 from $\left\{2,3\right\}$ and 1 from $\left\{1,4\right\}$ are respectively moved to 19 and 24. Comparing Figure 3 and Figure 4, one can find that this scheme thins the dense sensors and reduces the redundancy between the difference and sum sets. Furthermore, all the holes in the extended sparse ULA can be filled by the difference and sum results of the elements in $Z=\left\{4,9,14,19,24\right\}$ and the remaining dual pair elements 3, 4. Therefore, the newly formed array could have a larger co-array aperture than the NA. Although the scheme has the above advantages, it will result in some new holes in the original dense ULA part, e.g., holes 1 and 2 in Figure 4. Thus, in order to increase the DOF as large as possible, filling these new holes becomes an important issue that needs to be resolved when considering how to select the dual pair elements to move.

### 3.2. The Proposed Diff-Sum Nested Array

To resolve the hole issue caused by rearranging dual pair elements, a new array configuration known as DsNA is proposed. It achieves reduced redundancy. Meanwhile, all the holes in its physical array can be filled by the difference and sum results of sensors so that the virtual aperture is fully extended. The definition of the DsNA with $N_{1}+N_{2}$ sensors is given by:

**Definition** **2.** *(Diff-sum nested array). For two integers $N_{1}$ and $N_{2}$ satisfying $N_{1}\geq 3$ and $N_{2}\geq 2$, the diff-sum nested array is specified by the set $D_{DsNA}$, which is expressed as*

(14) $$\begin{array}{c} D_{DsNA}=\left\{\begin{array}{cc} X_{1}\cup Y_{1}\cup Z_{1}, & if\;N_{1}=2a-1,\;a\;is\;odd,\;a\geq 3,\\ X_{2}\cup Y_{2}\cup Z_{1}, & if\;N_{1}=2a-1,\;a\;is\;even,\;a\geq 4,\\ X_{2}\cup Z_{1}, & if\;N_{1}=2a-1,\;a\;is\;even,\;a<4,\\ \left\{0\right\}\cup X_{3}\cup Z_{3}, & if\;N_{1}=2a,\;a\geq 4,\\ \left\{0,1\right\}\cup X_{3}\cup {Z^{\prime }}_{3}, & if\;N_{1}=2a,\;a\leq 3, \end{array}\right. \end{array}$$

*where*

$$\begin{array}{c} X_{1}=\left\{2x\left|0\leq x\leq \left(N_{1}-1\right)/4\right.\right\},\\ Y_{1}=\left\{2x-1\left|\left(N_{1}+3\right)/4\leq x\leq \left(N_{1}-1\right)/2\right.\right\},\\ Z_{1}=\left\{N_{1}+n_{2}\left(N_{1}+1\right)\left|0\leq n_{2}\leq N_{2}-1+\left(N_{1}-1\right)/2\right.\right\},\\ X_{2}=\left\{2x\left|0\leq x\leq \left(N_{1}+1\right)/4\right.\right\},\\ Y_{2}=\left\{2x-1\left|\left(N_{1}+5\right)/4\leq x\leq \left(N_{1}-1\right)/2\right.\right\},\\ X_{3}=\left\{2x+1\left|1\leq x\leq \left(N_{1}-2\right)/2\right.\right\},\\ Z_{3}=\left\{N_{1}+n_{2}\left(N_{1}+1\right)\left|0\leq n_{2}\leq N_{2}-1+N_{1}/2\right.\right\},\\ {Z^{\prime }}_{3}=\left\{N_{1}+n_{2}\left(N_{1}+1\right)\left|0\leq n_{2}\leq N_{2}-2+N_{1}/2\right.\right\}. \end{array}$$

Note that Definition 2 is applicable to the conditions that $N_{1}\geq 3$ and $N_{2}\geq 2$, i.e., the sensor number satisfies R≥5. When $N_{1}=2a-1$, the total number of dual pairs is Ndual=N1+1N1+122 and the moved dual pair sensor number is Nc=N1−1N1−122. The moved sensors together with the original sparse ULA form the extended sparse ULA whose location set is $Z_{1}$. The retained sensors in the dense ULA locate at $X_{1}\cup Y_{1}$, $X_{2}\cup Y_{2}$ or $X_{2}$. When $N_{1}=2a\geq 8$, we have Nc=Ndual=N1N122. The location set of the extended sparse ULA is $Z_{3}$. The retained sensors of the dense ULA form the set $\left\{0\right\}\cup X_{3}$. When $N_{1}=2a<8$, Nc=N1N122−1 and Ndual=N1N122. The location set of the extended sparse ULA becomes ${Z^{\prime }}_{3}$, and the location set of the retained sensors of Subarray 1 becomes $\left\{0,1\right\}\cup X_{3}$.

**Figure 5:**
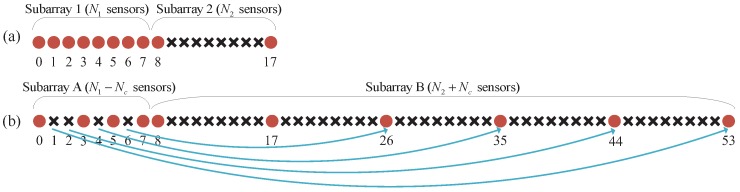
The configurations of (**a**) the nested array; and (**b**) the diff-sum nested array, where $\left(N_{1},N_{2}\right)=\left(8,2\right)$.

Figure 5 illustrates the relationship between the NA and the DsNA with $\left(N_{1},N_{2}\right)=\left(8,2\right)$. According to Proposition 2, the dual pairs of the NA are $\left\{1,8\right\}$, $\left\{2,7\right\}$, $\left\{3,6\right\}$ and $\left\{4,5\right\}$. The DsNA is constructed by moving 1, 2, 4 and 6 in these dual pairs to 26, 35, 44 and 53. Then, the dense ULA (Subarray 1 in Figure 5a) of the NA becomes a sparse array (Subarray A in Figure 5b) of the DsNA. The sparse ULA (Subarray 2 in Figure 5a) of the NA together with the moved sensors form an extended sparse ULA (Subarray B in Figure 5b) of the DsNA. Thus, the DsNA also consists of two subarrays. Subarray A is a sparse array containing nearly half the sensors in Subarray 1. Subarray B is a sparse ULA containing about $N_{1}/2$ more sensors than Subarray 2. Since the dense sensors are thinned, the redundancy and mutual coupling of the DsNA is much less than the NA.

Now, we will show that all the holes in the DsNA can be filled by the difference and sum results of sensors. For convenience, the consecutive range of any one virtual array in this paper is represented as $\left[-L_{c},L_{c}\right]$ with $L_{c}$ being the one-side DOF. The physical aperture is denoted by $L_{p}$, and is quantified as $L_{p}=\max\left\{D\right\}-\min\left\{D\right\}$. Since both the SS MUSIC method for constructing the DCa and the VCAM method for constructing the DSCa utilize the spatial smoothing method to solve the coherent issue generated by the Khatri–Rao product operation, the available DOF of the constructed virtual arrays in these methods would be reduced by half. Thus, the maximum number of detectable signals in these methods is equal to $L_{c}$. The following proposition gives some properties of the DSCa of the DsNA with $\left(N_{1},N_{2}\right)$.

**Proposition** **3.** *For the DsNA with $\left(N_{1},N_{2}\right)$, all the holes in its physical array can be filled by the difference and sum results of physical sensor locations. The one-side DOF of the DSCa can be expressed as*

(15) $$\begin{array}{c} L_{c}=\left\{\begin{array}{cc} N_{1}+\left(N_{1}+1\right)\left(N_{2}-1+\left(N_{1}-1\right)/2\right), & if\;N_{1}\;is\;odd,\\ N_{1}+\left(N_{1}+1\right)\left(N_{2}-1+N_{1}/2\right), & if\;N_{1}\;is\;even\;and\;N_{1}\geq 8,\\ N_{1}+1+\left(N_{1}+1\right)\left(N_{2}-2+N_{1}/2\right), & if\;N_{1}\;is\;even\;and\;N_{1}<8. \end{array}\right. \end{array}$$

**Proof.**  See Appendix B. ☐

**Figure 6:**
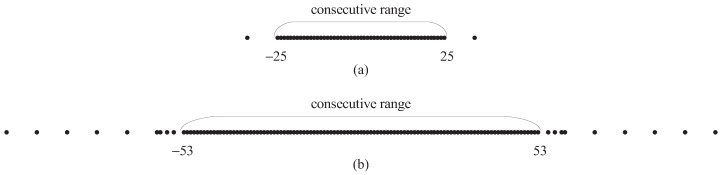
Two virtual configurations (R=10 and $\left(N_{1},N_{2}\right)=\left(8,2\right)$): (**a**) the DSCa of the NA; and (**b**) the DSCa of the DsNA.

Combining Definition 2 and Proposition 3, it is clear that, for case 1 and case 2, there exists the relationship $L_{c}=L_{p}$. For case 3, the relationship becomes $L_{c}=L_{p}+1$. Thus, Proposition 3 reveals the hole-free property of the DSCa of the DsNA in the range $\left[-L_{p},L_{p}\right]$. This means that the development of the DsNA can solve the hole issue caused by the dual pair element movement. Figure 6 shows the DSCa of the two configurations in Figure 5. One can find the DSCa of the DsNA contains all the consecutive elements in the range $\left[-53,53\right]$, where 53 is the physical aperture. However, the DSCa of the NA only comprises consecutive elements from −25 to 25.

To find out the optimum configuration in the DsNA set with a fixed sensor number $R=N_{1}+N_{2}$, we provide the following proposition. For convenience, the maximum value of $L_{c}$ is represented as $L_{cmax}$.

**Proposition** **4.** *When the sensor number R is fixed, the maximum one-side DOF of the DSCa has the following conclusions:*

**Proof.**  See Appendix C. ☐

According to Table 2, one can find that if R=8 or *R* is odd and R≥11, the optimal parameters are $N_{1}=R-3,N_{2}=3$. In the other cases, the consequences become $N_{1}=R-2,N_{2}=2$. For comparison, in Table 3, for the case of a large sensor number, we provide the approximate $L_{cmax}$ results of five virtual arrays, i.e, the DCa of the ANAII1 and ANAII2 [24], and the DSCa of the NA, CA and DsNA. For simplicity, we utilize DCa(ANAII1) and DCa(ANAII2) to represent the DCa of the ANAII1 and ANAII2. DSCa(NA), DSCa(CA) and DSCa(DsNA) represent the DSCa of the NA, CA and DsNA, respectively. The ANAII1 and ANAII2 are two kinds of arrays with the highest DOF among all the ANAs. Since [24] has only summarized the properties of the DCa of the ANA, we only consider the DCa of the these two arrays. From Table 3, we can conclude that DSCa(DsNA) has higher DOF than the other four virtual arrays. More DOF comparisons will be shown in the Simulation results.

**Remark** **2.** *In the detecting multiple pulsed radars application scenario, all kinds of sparse arrays can be used as the received array in the VCAM method for estimating directions of multiple deterministic orthogonal signals. However, traditional sparse arrays are usually developed based on the DCa concept so that they cannot take full advantage of aperture extending capacity of the DSCa concept. Therefore, the DSCa of the DsNA can always have higher DOF than that of sparse arrays developed by the DCa concept. In conclusion, in the detecting multiple pulsed radars application scenario, the DsNA is a more suitable sparse array.*

*It is noted that, in radar application, the secondary lobes height is one important beam pattern characteristic. However, the existing sparse array based beamformers are usually restricted to specific DCa based structures [13,31]. Only the DCa concept is used. Thus, using these beamformers to evaluate the secondary lobes height of the DsNA is not appropriate. In our future work, we will improve our VCAM method to obtain a DSCa based beamformer. In addition, for the DsNA, we will also analyze in the future about the trade-off strategy between its DOF and the secondary lobes height.*

## 4. Simulation Results

In this section, we evaluate the performances of five array configurations, i.e., the NA, CA, ANAII1, ANAII2, and DsNA. All the arrays have the same sensor number *R*. The parameters are optimal so that the maximum $L_{c}$ can be achieved.

### 4.1. DOF Comparison

**Figure 7:**
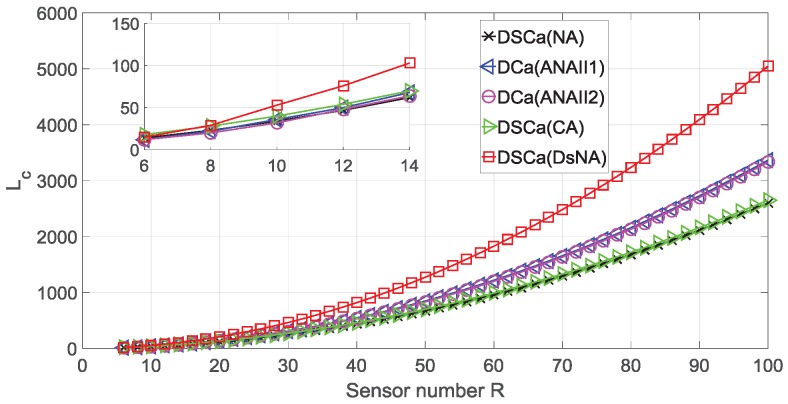
The values of $L_{c}$ of five virtual arrays with the sensor number *R* varying from 6 to 100.

As illustrated in Figure 7, the first experiment compares the values of $L_{c}$. The sensor number *R* varies from 6 to 100. It is obvious that the DSCa (DsNA) achieves a higher number of DOF than the other four virtual arrays. In addition, with the sensor number increasing, the gap between the DSCa (DsNA) and the other four virtual arrays becomes larger. In order to show the results more clearly, three examples with R=10, R=50 and R=90 are listed in Table 4. Combining Figure 7 and Table 4, we can conclude that the superiority of our proposed DSCa (DsNA) will be more obvious when the sensor number *R* grows larger.

### 4.2. DOA Estimation

**Figure 8:**
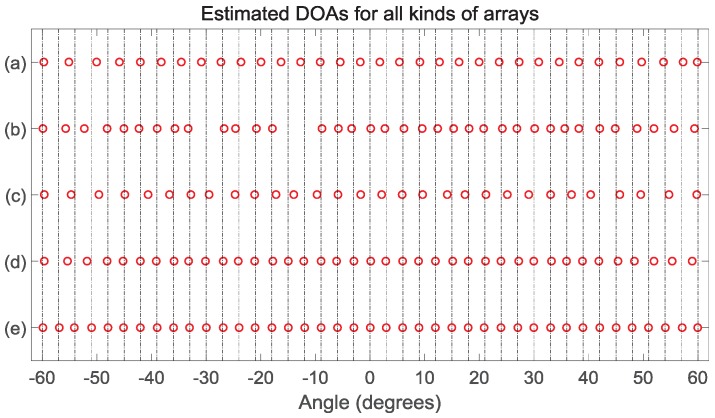
The estimated DOAs (R=10,Q=41 and SNR = 10 dB): (**a**) the NA with the VCAM used; (**b**) the ANAII1 with the SS MUSIC used; (**c**) the ANAII2 with the SS MUSIC used; (**d**) the CA with the VCAM used; and (**e**) the DsNA with the VCAM used.

As shown in Figure 8, the second experiment demonstrates the DOA estimations. The SS MUSIC method is applied to the ANAII1 and ANAII2. The VCAM method is applied to the NA, CA and DsNA. Here, we consider the sensor number of the five configurations as R=10 and the unit inter-element spacing as d=λ/2. The snapshots $N_{x}$ and the pseudo snapshots $N_{\tau }$ satisfy $N_{x}=N_{\tau }=800$. We consider the input SNR = 10 dB. Suppose there are 41 sources uniformly distributed between −60° and 60°. The frequencies of these 41 signals are uniformly distributed between 3 MHz and 10 MHz. Figure 8e shows that the DsNA can detect all the 41 sources since the one-side DOF of its DSCa is 53, which is shown in Table 4. However, as shown in Figure 8a–d, the other four configurations fail to detect all the DOAs correctly due to $L_{c}$ of the DSCa(NA), DCa(ANAII1), DCa(ANAII2), and DSCa(CA) being 34, 36, 32 and 40. Thus, with the sensor number *R* fixed, the DsNA with the VCAM used achieves a better performance than the other four array configurations.

### 4.3. Root Mean Square Error (RMSE)

In the third experiment, we conduct 500 Monte Carlo trials to evaluate the fidelity of the DOA estimations. The performance metric is the root mean square error (RMSE) of the DOA estimation, which is defined as

$$RMSE=\sqrt{\frac{1}{500Q}\sum_{i=1}^{500}\sum_{q=1}^{Q}{\left({\hat{\theta }}_{q}\left(i\right)-\theta_{q}\right)}^{2}},$$

where $\theta_{q}$ is the real DOA of the *q*th signal source and ${\hat{\theta }}_{q}\left(i\right)$ is the estimation for the *i*th trial, i=1,…,500. In this experiment, we consider the number of signal sources as Q=22. The sources are uniformly distributed between −60° and 60°, and their frequencies are uniformly distributed between 3 MHz and 10 MHz. The sensor number is R=10.

**Figure 9:**
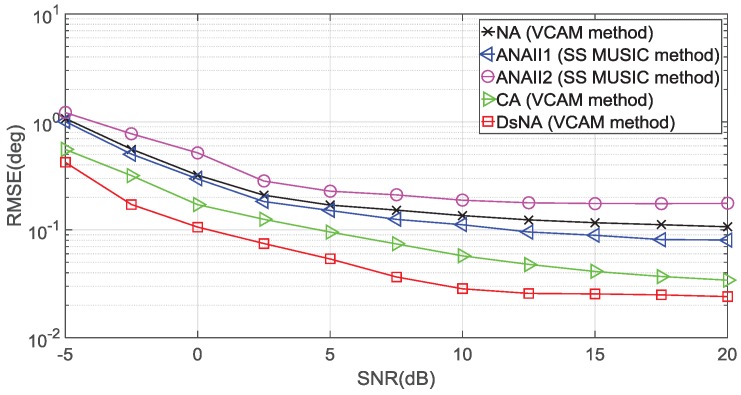
RMSE versus SNR, Q=22 and $N_{x}=N_{\tau }=800$.

In Figure 9, we suppose that $N_{x}=N_{\tau }=800$ to evaluate the RMSE performance as a function of the input SNR. It can be seen that the performances of the five configurations improve considerably when the SNR increases. The DsNA with the VCAM used outperforms the other four arrays since the DSCa(DsNA) contains more consecutive elements than the other four virtual arrays.

**Figure 10:**
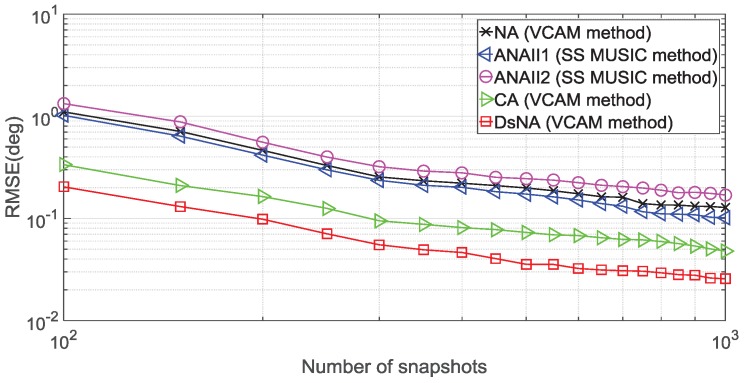
RMSE versus the number of snapshots, Q=22 and SNR = 10 dB.

In Figure 10, we set SNR = 10 dB and $N_{x}=N_{\tau }$ to evaluate the RMSE performance as a function of the number of snapshots. Similarly, the performances of all the configurations improve with the number of snapshots increasing. The DsNA with the VCAM used still performs better than the other four arrays, due to the larger consecutive range of its virtual array. From the two simulations, one can find that, even when the SNR and the number of snapshots are large, there is an obvious gap between the DsNA and the other four array configurations.

**Remark** **3.** *It is noted that the SS MUSIC method was originally designed for random signals. However, due to the similarity between the orthogonal characteristic of deterministic signals and the uncorrelated characteristic of random signals, the SS MUSIC method can also be used for DOA estimation of orthogonal deterministic signals. In this case, the statistical averaging for random signals in the original method is replaced by the time averaging for deterministic signals. As mentioned previously, the SS MUSIC method combines the KR product concept and the spatial information of received signals to construct the DCa. In contrast, the VCAM method jointly utilizes the KR product concept, spatial information and temporal information to obtain the DSCa with larger aperture than the DCa. The comparison between them may affect the future design of the sparse sensor array and improvement of co-array based methods for detecting multiple deterministic orthogonal signals.*

## 5. Conclusions

We have analyzed the properties of the DSCa of the NA and concluded that the DCa of the NA is a subset of its sum co-array. The redundancy between the two co-arrays would decrease the available DOF. Thus, by moving nearly half the sensors in the dense ULA of the NA to the right side of the sparse ULA, we proposed the DsNA whose DSCa has reduced redundancy, larger aperture and extended consecutive range than that of the NA. Compared with lots of novel sparse arrays such as the NA, ANA and CA, the DsNA achieves a higher number of DOF. The effectiveness of the proposed array was numerically studied and evaluated.

## Figures and Tables

**Table 1:** A summary of dual pairs of the NA with $\left(N_{1},N_{2}\right)=\left(4,3\right)$.

Hole Location	$d_{1D_{2}}-d_{1D_{1}}$	$d_{2D_{2}}+d_{2D_{1}}$	Dual Pair
5	9−4	4+1	$\left\{4,1\right\}$
6	9−3	4+2	$\left\{3,2\right\}$
7	9−2	4+3	$\left\{2,3\right\}$
8	9−1	4+4	$\left\{1,4\right\}$
10	14−4	9+1	$\left\{4,1\right\}$
11	14−3	9+2	$\left\{3,2\right\}$
12	14−2	9+3	$\left\{2,3\right\}$
13	14−1	9+4	$\left\{1,4\right\}$

**Table 2:** $L_{cmax}$ of the DSCa of the DsNA and the optimal parameters.

*R*	Optimal $N_{1}$, $N_{2}$	$L_{\mathrm{cmax}}$
odd (≥11)	$N_{1}=R-3,N_{2}=3$	$R^{2}/2+R/2-4$
odd (<11)	$N_{1}=R-2,N_{2}=2$	$R^{2}/2-3/2$
even (≥10)	$N_{1}=R-2,N_{2}=2$	$R^{2}/2+R/2-2$
8	$N_{1}=5,N_{2}=3$	29
6	$N_{1}=4,N_{2}=2$	15

**Table 3:** A summary of approximate $L_{cmax}$ of five virtual arrays.

Virtual Array	Approximate $L_{\mathrm{cmax}}$
DCa(ANAII1)	$R^{2}/3$
DCa(ANAII2)	$R^{2}/3$
DSCa(NA)	$R^{2}/4+R$
DSCa(CA)	$R^{2}/4+3R/2$
DSCa(DsNA)	$R^{2}/2+R/2$

**Table 4:** Examples of $L_{c}$ of the five virtual arrays.

Virtual Array	$L_{c}$ (R=10)	$L_{c}$ (R=50)	$L_{c}$ (R=90)
DSCa (NA)	34	674	2114
DCa (ANAII1)	36	848	2728
DCa (ANAII2)	32	832	2700
DSCa (CA)	40	700	2160
DSCa (DsNA)	53	1273	4093

## Abbreviations

The following abbreviations are used in this manuscript:

DSCa	difference and sum co-array
DOF	degree of freedom
NA	nested array
DsNA	diff-sum nested array
ULA	uniform linear array
DOA	direction of arrival
DCa	difference co-array
CA	coprime array
ANA	augmented nested array
VCAM	Vectorized Conjugate Augmented MUSIC
SS MUSIC	spatial smoothing based MUSIC
KR	Khatri–Rao
RMSE	root mean square error

## Appendix A

**Proof** **of** **Proposition** **2.** Since the location set of Subarray 2 of the NA is $D_{2}=\left\{N_{1}+n_{2}\left(N_{1}+1\right)\left|0\leq n_{2}\leq N_{2}-1\right.\right\}$, it is obvious that the holes in the NA form the set $D_{hole}=\left\{N_{1}+y\left(N_{1}+1\right)+x\left|0\leq y\leq N_{2}-2,1\leq x\leq N_{1}\right.\right\}$. According to Definition 1, one knows that for any dual pair $\left\{d_{1D_{1}},d_{2D_{1}}\right\}$ and any hole element $d_{h}\in D_{hole}$, one can have $d_{h}=N_{1}+\left(y+1\right)\left(N_{1}+1\right)-d_{1D_{1}}=N_{1}+y\left(N_{1}+1\right)+d_{2D_{1}}$, where $0\leq y\leq N_{2}-2$. Then, one can further have $d_{1D_{1}}+d_{2D_{1}}=N_{1}+1$. Thus, the closed form expression of dual pairs can be given as $P_{NA}=\left\{\left\{d_{1D_{1}},d_{2D_{1}}\right\}\left|1\leq d_{1D_{1}},d_{2D_{1}}\leq N_{1},d_{1D_{1}}+d_{2D_{1}}=N_{1}+1\right.\right\}$. Based on this expression, all the elements in $P_{NA}$ as well as the dual pair number can be easily obtained as summarized in Proposition 2. ☐

## Appendix B

**Proof** **of** **Proposition** **3.** We will first prove that all the holes in the DsNA can be filled by the difference and sum results of sensors, i.e., $L_{ds}$ has no holes in the range $\left[-L_{p},L_{p}\right]$. It is noted that, since $L_{ds}$ is symmetrical with the zero point as the center, the holes in the flipped array of the DsNA would also be filled. According to the dual characteristic of the NA, Proposition 2 and Definition 2, it is obvious that no matter if $N_{1}$ is odd or even, all the holes in the sparse ULA (Subarray B) of the DsNA can be filled by the difference and sum results. Then, we only need to prove that the holes in the range $\left[0,N_{1}-1\right]$ of the DsNA can be filled. If $N_{1}$ and $(N_{1}+1)/2$ are odd, the location set of Subarray A is $D_{A}=X_{1}\cup Y_{1}$. As $Y_{1}-X_{1}=\left\{2x-1\left|1\leq x\leq \left(N_{1}-1\right)/2\right.\right\}$ and $X_{1}+X_{1}=\left\{2x\left|0\leq x\leq \left(N_{1}-1\right)/2\right.\right\}$, it can be seen that $\left\{Y_{1}-X_{1}\right\}\cup \left\{X_{1}+X_{1}\right\}$ contains all the elements in the range $\left[0,N_{1}-1\right]$. If $N_{1}$ is odd and $(N_{1}+1)/2$ is even: with $N_{1}\geq 7$, $D_{A}=X_{2}\cup Y_{2}$. Due to $Y_{2}-X_{2}=\left\{2x-1\left|1\leq x\leq \left(N_{1}-1\right)/2\right.\right\}$ and $X_{2}+X_{2}=\left\{2x\left|0\leq x\leq \left(N_{1}+1\right)/2\right.\right\}$, it can be seen that $\left\{Y_{2}-X_{2}\right\}\cup \left\{X_{2}+X_{2}\right\}$ contains all the elements in the range $\left[0,N_{1}-1\right]$. With $N_{1}<7$, i.e., $N_{1}=3$, $D_{A}=X_{2}=\left\{0,2\right\}$. It is obvious that $\left\{N_{1}-X_{2}\right\}\cup \left\{X_{2}+X_{2}\right\}$ contains all the elements in the range $\left[0,N_{1}-1\right]$. If $N_{1}$ is even: With $N_{1}\geq 8$, $D_{A}=\left\{0\right\}\cup X_{3}$. According to Definition 2, we obtain $\left\{X_{3}-X_{3}\right\}=\left\{2x\left|0\leq x\leq \left(N_{1}-4\right)/2\right.\right\}$ and $\left\{X_{3}-0\right\}=\left\{2x-1\left|2\leq x\right.\right.\left.\left.\leq N_{1}/2\right.\right\}$. Then, $\left\{X_{3}-X_{3}\right\}\cup \left\{X_{3}-0\right\}$ contains not only all the consecutive lags in $\left[2,N_{1}-3\right]$ but also 0 and $N_{1}-1$. One can be obtained by the difference result of $N_{1}$ in $Z_{3}$ and $N_{1}-1$ in $X_{3}$. $N_{1}-2$ can be obtained by the sum result of $N_{1}/2$ and $N_{1}/2-2$ in $X_{3}$ when $N_{1}/2$ is odd. When $N_{1}/2$ is even, $N_{1}-2$ can be obtained by the sum result of $N_{1}/2-1$ and $N_{1}/2-1$ in $X_{3}$. Thus, $L_{ds}$ contains all the elements in the range $\left[0,N_{1}-1\right]$. When $N_{1}\leq 6$, $D_{A}=\left\{0,1\right\}\cup X_{3}$. Here, the reason of retaining 1 is that when $N_{1}=6$, we have $N_{1}/2-2=1$, and when $N_{1}=4$, we have $N_{1}/2-1=1$. Therefore, $N_{1}-2$ can still be obtained by the sum result. In conclusion, $L_{ds}$ has no holes in the range $\left[-L_{p},L_{p}\right]$. Then, we will calculate the value of $L_{c}$. The physical aperture $L_{p}$ is the maximum element in the location set so that $L_{p}$ is always contained in $D_{DsNA}$. From the configuration of the DsNA, it is obvious that, if $L_{ds}$ contains $L_{p}+1$ and $-(L_{p}+1)$, these two elements must be obtained by the sum operation of 1 and $L_{p}$. According to Definition 2, we know that if $N_{1}$ is odd, 1 is not contained in $D_{DsNA}$. Thus, $L_{p}+1$ and $-(L_{p}+1)$ are holes in $L_{ds}$ and we have $L_{c}=L_{p}=N_{1}+\left(N_{1}+1\right)\left(N_{2}-1+\left(N_{1}-1\right)/2\right)$. Similarly, if $N_{1}$ is even and no less than 8, 1 is not contained in $D_{DsNA}$ either so that $L_{c}=L_{p}=N_{1}+\left(N_{1}+1\right)\left(N_{2}-1+N_{1}/2\right)$. If $N_{1}$ is even and less than 8, 1 is contained in $D_{DsNA}$ but 2 is not contained so that $L_{c}=L_{p}+1=N_{1}+1+\left(N_{1}+1\right)\left(N_{2}-2+N_{1}/2\right)$. ☐

## Appendix C

**Proof** **of** **Proposition** **4.** Since $R=N_{1}+N_{2}$ is a given sensor number, we can turn $L_{c}$ in the three cases in Proposition 3 into three functions: (case 1) If $N_{1}$ is odd, $f_{1}\left(N_{1}\right)=N_{1}+\left(N_{1}+1\right)\left(R-N_{1}-1+\left(N_{1}-1\right)/2\right)$. (case 2) If $N_{1}$ is even and $N_{1}\geq 8$, $f_{2}\left(N_{1}\right)=N_{1}+\left(N_{1}+1\right)\left(R-N_{1}-1+N_{1}/2\right)$. (case 3) If $N_{1}$ is even and $N_{1}<8$, $f_{3}\left(N_{1}\right)=N_{1}+1+\left(N_{1}+1\right)\left(R-N_{1}-2+N_{1}/2\right)$. The derivatives of the three functions are respectively $\partial f_{1}/\partial N_{1}=R-1-N_{1}$, $\partial f_{2}/\partial N_{1}=R-1/2-N_{1}$ and $\partial f_{3}/\partial N_{1}=R-3/2-N_{1}$. Since $N_{1}\leq R-2$, the three derivatives are more than 0. Thus, when considering only one of the three cases, the maximum $L_{c}$ is achieved with $N_{1}$ being maximum. Now, we would further compare the maximum $L_{c}$ of the three cases. If R is odd and R<11, case 1 and case 3 would be compared. With $N_{1}=R-2$ and $N_{2}=2$, the maximum $L_{c}$ of case 1 can be achieved, i.e., $f_{1}\left(R-2\right)=R^{2}/2-3/2$. When $N_{1}=R-3$ and $N_{2}=3$, the maximum $L_{c}$ of case 3 can be achieved, i.e., $f_{3}\left(R-3\right)=R^{2}/2-R/2-1$. As R≥5, we have $f_{1}\left(R-2\right)-f_{3}\left(R-3\right)>0$. Thus, in this case, we have $L_{cmax}=R^{2}/2-3/2$ and the optimal parameters are $N_{1}=R-2$ and $N_{2}=2$. If R is odd and R≥11, case 1 and case 2 would be compared. The maximum $L_{c}$ of case 1 is still $f_{1}\left(R-2\right)=R^{2}/2-3/2$. With $N_{1}=R-3$ and $N_{2}=3$, the maximum $L_{c}$ of case 2 can be achieved, i.e., $f_{2}\left(R-3\right)=R^{2}/2+R/2-4$. Then, we can have $f_{1}\left(R-2\right)-f_{2}\left(R-3\right)<0$. Thus, $L_{cmax}=R^{2}/2+R/2-4$ and the optimal parameters are $N_{1}=R-3$ and $N_{2}=3$. If R is even and R≥10, case 1 and case 2 would be compared. With $N_{1}=R-3$ and $N_{2}=3$, the maximum $L_{c}$ of case 1 can be achieved, i.e., $f_{1}\left(R-3\right)=R^{2}/2-3$. With $N_{1}=R-2$ and $N_{2}=2$, the maximum $L_{c}$ of case 2 can be achieved, i.e., $f_{2}\left(R-2\right)=R^{2}/2+R/2-2$. It is obvious that $f_{2}\left(R-2\right)>f_{1}\left(R-3\right)$. Thus, in this case, we have $L_{cmax}=R^{2}/2+R/2-2$ and the optimal parameters are $N_{1}=R-2$ and $N_{2}=2$. If R is even and R<10, case 1 and case 3 would be compared. The maximum $L_{c}$ of case 1 is still $f_{1}\left(R-3\right)=R^{2}/2-3$. With $N_{1}=R-2$ and $N_{2}=2$, the maximum $L_{c}$ of case 3 can be achieved, i.e., $f_{3}\left(R-2\right)=R^{2}/2-R/2$. Then, we have $f_{1}\left(R-3\right)-f_{3}\left(R-2\right)=R/2-3$. It is obvious that when R≤6, $f_{3}\left(R-2\right)\geq f_{1}\left(R-3\right)$. In this case, we have $L_{cmax}=R^{2}/2-R/2$ and the optimal parameters are $N_{1}=R-2$ and $N_{2}=2$. When R=8, we have $f_{3}\left(R-2\right)<f_{1}\left(R-3\right)$. In this case, we have $L_{cmax}=R^{2}/2-3$ and the optimal parameters are $N_{1}=R-3$ and $N_{2}=3$. ☐
